# Female genital mutilation/cutting incidence, diagnostic capacities, and obstetric outcomes among migrant women: a single-center retrospective analysis in a 10-year birth cohort in Austria

**DOI:** 10.1186/s12889-022-14773-7

**Published:** 2023-01-10

**Authors:** N. Taumberger, T. Gruber, K. Edler, G. Trutnovsky, T. Bracic, N. Semrl, A.-M. Schütz, K. Eisnecker, K. Tamussino, H. Fluhr

**Affiliations:** Department of Obstetrics and Gynecology, Medical University of Graz, Auenbruggerplatz 14, 8036 Graz, Austria

**Keywords:** Female genital mutilation/cutting, Prevalence, Austria, Indirect estimates, Diagnostic capacities

## Abstract

**Introduction:**

Practical experience in the care of women with female genital mutilation/cutting (FGM/C) is uncommon in Austria. However, affected women require specialized gynecological and obstetric care. In our region, there is currently neither an official counseling center nor specially trained medical personnel to address the special needs of women after FGM/C. The aim of this study was to determine the potential need for obstetric care for women who have undergone FGM/C in our region.

**Methods:**

We retrospectively reviewed women presenting for delivery at the LKH University Hospital Graz from 1.1.2010 until 31.12.2020 regarding the place of birth and/or the nationality of the mother to filter out women from a country with known FGM/C prevalence according to the UNICEF Global Database. Data on the documentation of FGM/C as well as demographic maternal data and peripartal parameters were gathered. Periods before and after the European refugee crisis in 2015 were compared.

**Results:**

During the study period, a total of 35,628 deliveries took place at our hospital. 856 (2.4%) deliveries of 539 women were included due to nationality or birthplace in a country with known FGM/C prevalence. We found only 17/539 (3.2%) documented FGM/C cases. The estimated FGM/C prevalence among those patients was, however, 208/539 (38,6%). Women affected by FGM/C in our collective were most frequently from Nigeria, Egypt, Iraq, Ghana, and Somalia. No statistically significant increase in deliveries during the study period in the overall study cohort was observed, with the exception of deliveries of Somali women *(p = 0.000).*

**Discussion:**

The discrepancy between documented and expected FGM/C rates (3,2% vs. 38,6%) in our collective suggests that most cases of FGM/C go undetected among women delivering in Austria. These data show the great need for special training for obstetricians and targeted contact points for affected women.

## Background

Female genital mutilation/cutting (FGM/C) is defined as “all procedures that involve the partial or total removal of external genitalia or other injury to the female genital organs for non-medical reasons” [1]. FGM/C affects at least 200 million women and girls worldwide and it is estimated that each year, around 3.6 million women and girls are at risk of FGM/C [2, 3]. The World Health organization (WHO) differentiates four types of FGM/C: Type I, the partial or total removal of the clitoral glans, Type II, additional excision of the labia minora, Type III, also known as “infibulation”, that is narrowing of the vaginal opening after removing the labia minora and majora with or without the clitoral glans, and Type IV, which is classified as “all other harmful procedures to the female genitalia for non-medical purposes, for example pricking, piercing, incising, scraping and cauterization “ [4, 5]. Type IV FGM/C is more common in the developed world but often not referred to as FGM/C and seen as a cosmetic procedure when it comes to genital piercings. The United Nations (UN) and the European Union (EU) have initiated the global “Spotlight Initiative”. In 2018 they announced a joint goal of eliminating FGM/C by year 2030 [6].

FGM/C has no health benefits and can cause immediate and long-term health problems [1] including genitourinary [7–12] and obstetrical complications [9, 13–15], sexual health [4, 8, 9, 16–18], psychological problems [18, 19], and infection [8, 9, 20]. Girls and women suffering from or living with FGM/C require quality and knowledgeable health care [1, 4].

FGM/C is practiced predominantly in sub-Saharan Africa and the Arab States, but also in many other regions throughout Africa, Asia, Eastern Europe and Latin America [21, 22]. Comprehensive surveys [23] have estimated the prevalence of FGM/C in countries featuring a high prevalence of practice can be as high as 98% [24]. The highest prevalence of FGM/C among girls under the age of 14 years is reported in Gambia (around 56%), Mauritania (around 54%) and Indonesia (around 50%) whereas the highest prevalence in females between 15 and 49 years is reported in Somalia (98%), Guinea (97%) and Djibouti (93%) [3, 24]. Representative data have been collected in 27 African and 3 Asian countries [23, 25]. However, FGM/C also takes place in other countries, but representative data are lacking.

Migration of affected females to high resource countries such as United States, Australia and Europe makes FGM/C a global concern [2]. An estimated 578,000 first-generation women and girls affected by FGM/C were living in the European Union, Norway, and Switzerland in 2011 [26]. Demographic projections indicate that between 2016 and 2030 more than 400,000 women and girls being affected by FGM/C before migration will arrive in the EU28 member states. This means that the number of women and girls living with FGM/C and seeking healthcare will further increase in the coming years in many middle- and high resource countries [27].

On 01.01.2022, there were 4,553,444 women living in Austria. Among them approximately 628,381 were girls under the age of 15 [28]. The exact prevalence of FGM/C in Austria is currently unknown. In 2019 the European Institute for Gender Equality estimated that 735–1083 girls under the age of 18 residing in Austria are at risk of FGM/C, which is a remarkable number [29]. FGM/C is not included in the educational curricula of the Austrian health worker force and experience with FGM/C-related care, including recording and reporting of FGM/C is scarce. Only a limited number of FGM/C outpatient clinics attached referral hospitals (three in Vienna and one in Tyrol) provide counseling, support and reconstructive treatment such as defibulation [29, 30]. Furthermore, adverse obstetric outcomes have been reported and recent studies suggest that women, subjected to FGM/C have a higher risk for caesarean section, higher degree perineal tear, episiotomy and postpartum hemorrhage [31–33].

However, obstetrician and midwifes lack the proper training. Establishment of FGM/C outpatient clinics across the country would be an important step towards developing a comprehensive, national action plan to address FGM/C related issues, offer education for health care professionals, provide specialized health services and therefore improve trust between health practitioners and FGM/C affected communities as proposed by the European Institute for Gender Equality [29].

This retrospective analysis assessed the number of women from FGM/C practicing countries admitted to the labor ward of the Department of Obstetrics and Gynecology, Medical University of Graz, Graz, Austria between 2010 and 2020. The expected number of patients affected by FGM/C was estimated based on the prevalence in the country of origin and compared to the actual number of documented FGM/C cases. The aim was to assess the number of women in the catchment area of our health care facility who originally migrated from FGM/C practicing countries. We assumed that our analysis would provide insights into the diagnostic and recording capacities of the university hospital’s healthcare providers and raise awareness among health care professionals. The secondary objective was to compare obstetric outcomes of women subjected to FGM/C with general population in our region.

## Material and methods

We retrospectively reviewed hospital records of women coming from one of the 30 FGM/C practicing countries [24] that gave birth at our referral hospital between January 2010 and December 2020. The study was approved by the ethics committee of the Medical University of Graz under the number: EK Nr: 1675/2020.

A total of 35,628 deliveries took place during this time period. Records of all women coming from FGM/C practicing countries were screened for potential documentation of FGM/C, maternal characteristics as well as mode of delivery and the postpartum course. Screened maternal data included nationality, country of birth, age at delivery, and time of first visit to the hospital (delivery or antenatal). Antenatal records from our outpatient clinic, delivery protocols written by doctors and midwifes in our obstetric electronic medical chart (PIA) and discharge letters were screened for documentation of FGM/C and/or defibulation, either as a coded or descriptive diagnosis. We also extracted information on the mode of delivery, duration of first and second stage of labor, analgesia, episiotomy, defibulation, complications during delivery and potential language barrier.

The primary outcome was the number of women from FGM/C practicing countries presenting for delivery at our hospital and the number of documented FGM/C cases. Secondary outcomes were the obstetrical outcomes and change in size of the patient collective before and after the start of the refugee crisis in 2015.

The most recent estimated country prevalence of FGM/C of women aged 15–49 (except Indonesia: aged 0–11) [2, 34] were used to estimate the number of women after FGM/C in our patient collective. The absolute number of women with the same country of birth/nationality was multiplied by the corresponding country prevalence estimates to calculate the number of women where FGM/C could have been present. The estimate was compared to the actual number of documented FGM/C.

IBM SPSS Statistics 27 software was used to provide descriptive statistics.

Obstetric data of the study cohort were compared to the overall obstetric data of the region [35].

## Results

### Primary results

**Fig. 1:**
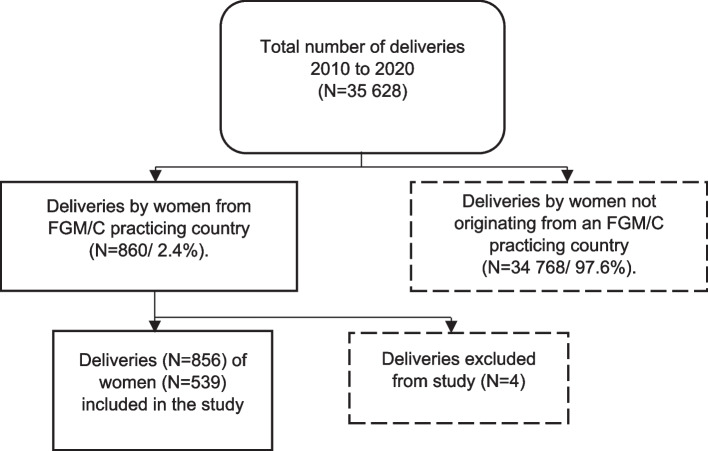
Flow Chart of patient selection

Amongst the 35.628 deliveries in the 10-year study period, 860 (2.4%) were by women with a nationality and/or country of birth with a known FGM/C prevalence. After exclusion of four deliveries because of missing data, 856 deliveries of 539 women remained for the analysis as shown in Fig. 1.

**Fig. 2:**
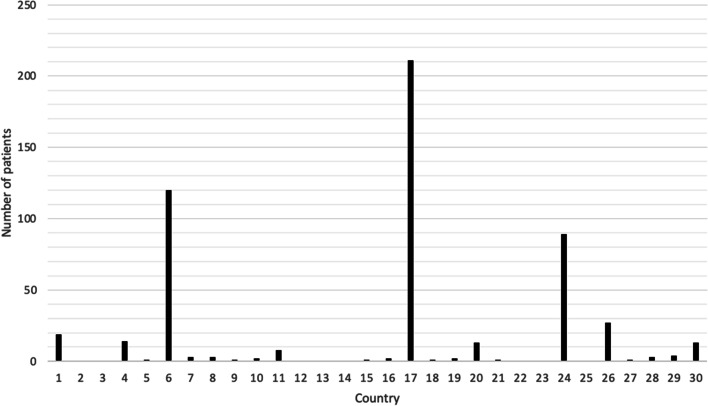
Number of patients at our hospital from the 30 countries included with known prevalence data of FGM/C; 1. Somalia (*N* = 19), 2. Guinea (*N* = 0), 3. Djibouti (*N* = 0), 4. Sierra Leone (*N* = 14), 5. Mali (*N* = 1), 6. Egypt (*N* = 120), 7. Sudan (*N* = 3), 8. Eritrea (*N* = 3), 9. Burkina Faso (*N* = 1), 10. Gambia ( *N* = 2), 11. Ethiopia (*N* = 8), 12. Mauritania (*N* = 0), 13. Liberia (*N* = 0), 14. Guinea-Bissau (*N* = 0), 15. Chad (*N* = 1), 16. Ivory Coast (*N* = 2), 17. Nigeria (*N* = 211), 18. Senegal (*N* = 1), 19. Central African Republic (*N* = 2), 20. Kenya (*N* = 13), 21. Yemen (*N* = 1), 22. United Republic of Tanzania (*N* = 0), 23. Benin (*N* = 0), 24. Iraq (*N* = 89), 25. Togo (*N* = 0), 26. Ghana (*N* = 27), 27. Niger (*N* = 1), 28. Uganda (*N* = 3), 29. Cameroon (*N* = 4), 30. Indonesia (*N* = 13)

In our collective, the largest group of patients were from Nigeria (*n* = 211), Egypt (*n* = 120), Iraq (*n* = 89), Ghana (*n* = 27) and Somalia (*n* = 19) *(*Fig. 2*)*. For all years combined, 17 women had a coded or descriptive diagnosis of FGM/C. The estimated prevalence of FGM/C was compared to the actual number of documented FGM/C for each high-risk country (Table 1). There was a substantial difference between documented cases and estimated prevalence for all patients from high-risk countries. The expected number of women affected by FGM/C was approximately 208/539 (38,6%), however, only 17/539 (3,2%) cases were documented. This suggests that in 191 (92%) cases FGM/C might have been overseen.

**Table 1:** Documented and estimated prevalence of FGM/C in women from high-risk countries presenting at the University clinic Graz between 2010 and 2020

Country	Patients (N)	documented FGM/C cases	FGM/C country prevalence (23,25)	Expected FGM/C cases
Nigeria	211	6 (2,2%)	19%	40
Egypt	120	0 (0%)	87%	104
Iraq	89	0 (0%)	7%	6
Ghana	27	0 (0%)	4%	1
Somalia	19	8 (42,1%)	98%	19
Sierra Leone	14	0 (0%)	86%	12
Indonesia	13	0 (0%)	49%	6
Kenya	13	1	21%	3
Ethiopia	8	0 (0%)	65%	5
Cameroon	4	0 (0%)	1%	0
Eritrea	3	0 (0%)	83%	3
Sudan	3	1	87%	3
Uganda	3	0 (0%)	0%	0
Central African Republic	2	0 (0%)	24%	1
Gambia	2	1	76%	2
Ivory Coast	2	0 (0%)	37%	1
Burkina Faso	1	0 (0%)	76%	1
Chad	1	0 (0%)	38%	0
Mali	1	0 (0%)	89%	1
Niger	1	0 (0%)	2%	0
Senegal	1	0 (0%)	24%	0
Yemen	1	0 (0%)	19%	0
**Total**	**539**	**17**		**208**

Among 856 deliveries of the 539 women included in the study, there were 836 singletons, 19 twin and one triplet pregnancy, resulting in 877 born children.

### Secondary results

The median as well as the mean age at the time of delivery was 31 years with a range from 14 to 41 years (Table 2). The median gravidity was 3 with a range from 1 to 12 and the median parity was 3 with a range from 1 to 9 (Table 3).

**Table 2:** Age of the patient cohort

Age at delivery	Numbers	Percent
under 18	5	0,60%
18–29	339	39,60%
30–34	262	30,60%
35–39	197	23%
over 40	53	6,20%

**Table 3:** Number of pregnancies and parity of the patient cohort

	Minimum	Maximum	Average	Median
**Gravidity**	1	12	3,3	3,0
**Parity**	1	9	2,7	3,0

51.2% of the deliveries included in the study occurred between 1.7.2015 and 31.12.2020, after the beginning of the European refugee crisis. An increase in deliveries after 1.7.2015 was documented by women from 5 countries with the highest FGM/C prevalence rates at our hospital.

However, only the increase in women from Somalia was statistically significant *(p = 0.000)* (Table 4).

**Table 4:** Increase in deliveries between study periods

Periode		1.1.2010–30.6.2015	1.7.2015–31.12.2020	
Country of origin	FGM/C prevalence from february 2020 (24)	N	N	p
**Somalia**	**98%**	**1**	**23**	***0.000***
Sudan	87%	2	3	*1.000*
Sierra Leone	86%	5	10	*0.302*
Eritrea	83%	0	4	*Not applicable*
Gambia	76%	1	2	*1.000*

Demographic data are shown in Table 5.

**Table 5:** Demographic data of the patient cohort

	Numbers	Percent
**Study cohort**		
Deliveries in total	856	100%
Patients	539	100%
**First contact**		
Prepartum	777	9%
During birth	79	91%
**Notation of FGM/C at delivery**		
Yes	22	2,6%
No	834	97,4%
**Defibulation before delivery**		
Yes	3	0,6%
No	536	99,4%
**Defibulation during delivery**		
Yes	0	0%
No	856	100%
**Infectious diseases of the collective**		
Hepatitis B	16	3%
Hepatitis C	6	1,1%
HIV	9	1,7%
Tuberculosis	2	0,4%
CMV	7	1,3%
Gonorrhea	1	0,2%

0.6% of the women had a defibulation documented before delivery and none during delivery. Given the known and estimated prevalence of FGM/C in those countries [24], we would have expected a rate of 38,6% in this collective, i.e., about 208 women affected by FGM/C. This suggests we missed or did not document most cases of FGM/C. 91% of women had their first contact with our department before time of delivery.

A language barrier was explicitly noted in 17% (*n* = 146) out of 856 deliveries.

Table 6 shows the mode of delivery, peripartal complications and episiotomy rate. Compared to the “local labor and delivery register data 2019” which included all women giving birth in 2019 in our hospital, the rate of vaginal deliveries in the study cohort was almost the same, 55% vs. 56%, respectively. The rate of assisted vaginal deliveries was 4.9% vs. 7.2%, and the rate of cesarean section was 40.1% vs 36.4%, respectively. The rate of primary cesarean sections in the study cohort was 22% compared to 18.1% in the comparative collective. The episiotomy rate was 12.3% vs. 18.4%, respectively.

**Table 6:** Mode of delivery and peripartal parameters

	Numbers	Percent
**Mode of delivery**		
spontaneous vaginal	471	55.0%
Assisted vaginal	42	4.9%
Vacuum extraction	39	4.6%
Forceps	2	0.2%
Breech delivery	1	0.1%
Cesarean section	343	40.1%
primary	191	22.3%
secondary	152 (77 fetal/75 maternal)	17.8%
**Episiotomy**		
Yes	63	12.3%
At spontaneous delivery	33	6.4%
At assisted vaginal delivery	30	5.8%
No	450	87.7%
**Complications**		
Perineal tear 3rd/4th degree	6	1.2%
Intrapartal hemorrhage	1	0.2%
Birth injury explicitly due to FGM/C	6	1.2%
Clitoral Tear	12	2.3%
of which with FGM/C documentation	6	1.2%
of which without FGM/C documentation	6	1.2%
paraclitoral labial laceration	4	0.8%
of which with FGM/C documentation	1	0.2%
of which without FGM/C documentation	3	0.6%

## Discussion

Our analysis retrospectively assessed the number of women from a country where FGM/C is practiced giving birth in our region. In 10-year period 539 women from high-risk countries delivered at our center and among them, 17 (3.2%) cases of FGM/C were documented. This was significantly less than what was expected based on prevalence estimates (*n* = 208; 38,6%). Our results suggest most FGM/C cases are not diagnosed and/or documented. Lack of awareness and incorrect documentation have been described in other studies undertaken in Europe and worldwide [36–40].

A recent study from Switzerland looked at female patients from one of the 30 countries in which FGM/C is practiced hospitalized from 2016 to 2018. FGM/C was coded in 2.3% of cases. However, using indirect estimation methods an FGM/C prevalence of 57% was expected. Thus, in about 96% of patients estimated to be affected by FGM/C, the condition was not adequately identified, documented, and/or coded [41], which is consistent with our findings. Knowledge gaps among health workers regarding the prevalence, diagnosis, and management of FGM/C, especially in high-income countries are well documented [36, 37, 42–48].

Although the lack of awareness among health workers seems to play a major role in under-recording of FGM/C, other factors may be responsible for a drastic difference in estimated and actual numbers of detected FGM/C cases. Firstly, the extrapolation from the estimated FGM/C prevalence published by UNICEF does probably not reflect the reality in the population of migrant women living in Austria. Support for the FGM/C practice among migrants from FGM/C practicing countries coming to regions with low or no FGM/C prevalence may decrease, especially after residing in a country where FGM/C is illegal for a longer period of time. Furthermore, migrants who are not supportive of the practice may be more likely to migrate to a non-practicing country [41, 49, 50]. Studies suggest that immigrants tend to be younger and more educated, thus more likely to understand the consequences of FGM/C [51]. This selective migration likely contributes to the difference between expected and actual detected numbers of FGM/C.

Secondly, the appearance after FGM/C varies greatly with parity, age, and type of practice. FGM/C might be clinically difficult to recognize even when health workers are aware of the risk [52].

Increase in the number of deliveries between 1.7.2015–31.12.2020 when compared to the time period between 1.1.2010–30.6.2015 was documented by women from 5 out of 10 countries with the highest FGM/C prevalence rates. These are Somalia (with FGM/C prevalence of 98%), Sudan (87%), Sierra Leone (86%), Eritrea (83%), and Gambia (76%). Given the high prevalence of FGM/C among women coming from these countries, obstetric personnel should be especially vigilant for possible female genital mutilation among this collective.

Although no significant increase in overall deliveries by women coming from FGM/C practicing countries could be demonstrated, it can be expected that the number of births by women with FGM/C will increase across Europe and thus also in Austria and Graz. This assumption is confirmed by a demographic forecast which predicts that the EU28 countries will welcome about 1.3 million migrant women from FGM/C practicing countries between 2016 and 2030. About one-third of these migrant women are expected to have already been affected by FGM/C prior to immigration [27].

Expected increases may expose healthcare professionals to health risks of FGM/C, defined by WHO [1]. Poor communication between affected women and healthcare workers, cultural differences, language barrier, lack of training and fear of consultation need to be addressed.

Language barriers make it difficult for women to understand treatment protocols and health information or making shared decisions [53]. Our data reveals that in 17% (*n* = 146) of 856 deliveries an existing language barrier was explicitly noted. The WHO guidelines for treating women with FGM/C state that in case of a language barrier an official interpreter should be consulted [1]. Interpreters should be qualified and preferably female, not a family member or a friend of the affected person, and, in the best case scenario, familiar with FGM/C [4]. In everyday practice and especially in a busy labor ward situation, however, this is often not practicable. This illustrates once more the need for specialized consultation-hours where best possible counseling and treatment can be offered beforehand. There, affected women can receive interdisciplinary care with psychologists, interpreters and trained health professionals working together.

Sociocultural differences complicate healthcare for women with FGM/C further. Some girls and women feel shame and anger when they were labeled as “different” and “mutilated” by host country health workers and felt culturally misunderstood. This may lead to concealment and feeling extremely uncomfortable during medical examinations. Open communication and mutual understanding between those affected and the healthcare personnel treating them can subsequently be very difficult to achieve [54].

Awareness-raising and training of healthcare staff can improve documentation and identification of FGM/C. Following such efforts, including the awareness-raising and the implementation of training programs, 18 Belgian hospitals detected the number of documented FGM/C cases - compared to the median number in previous years – to increase by a factor of 2.5, resulting in a significant increase in the perceptiveness of the issue [38].

Appreciation and recognition of FGM/C is particularly important in obstetrics and perinatal care [9, 13–15]. Although previous studies showed that the risk of episiotomies is increased in women with FGM/C compared to women without FGM/C [9, 13, 14], the episiotomy rate was lower in our study population (12.3%) compared to the overall episiotomy rate in our region (18.6%) [35]. The rate of cesarean deliveries in the overall cohort (36.4%) was slightly lower than that of the study population (40.1%), but not statistically significant. However, the rate of primary cesarean sections in the study population (22.3%) was significantly higher compared to overall rate of primary cesarean deliveries in our region (18.1%). A potentially increased risk of cesarean delivery among women with FGM/C is especially important due to a higher fertility rate in this collective. In our study population, the mean parity of 2.7 is significantly higher than the fertility rate of 1.44 per women in Austria [55]. Thus, the decision whether to perform a cesarean section in primiparous women with FGM/C should be made with particular care, especially in view of the increased likelihood of subsequent pregnancies and the associated increased morbidity.

Finally, with an estimated number of 2.6 births per month by women who have undergone female genital mutilation and the assumption that approximately 50% of the children born are female, it can be estimated that every month at least one girl is born in our hospital who is at risk of becoming a victim of FGM/C in the future. To protect further generations from the performance of FGM/C, it is of great importance that the presence of FGM/C is detected by the health personnel. The parents of newborn daughters should be informed and counseled about the legal situation in Austria regarding FGM/C and the importance of physical integrity for healthy child development should be emphasized [56]. These preventive measures should also be continued by the future pediatrician, which once again points to the importance of interdisciplinary collaboration in this special patient population [30]. The WHO and other institutions provide educational resources which can be referenced for guidance [1, 4, 28].

### Strengths

To date, this retrospective data analysis is the first to address the issue of FGM/C in an Austrian hospital. Our study included a large sample size at an Austrian tertiary center and covered a long study period (2010–2020). The study period was chosen to allow a comparison of deliveries before the wave of refugees in 2015 (1.1.2010–30.6.2015) with the period after (1.7.2015–31.12.2020) and consequently analyze potential effects of the refugee crisis on the number of births by women from FGM/C practicing countries.

### Limitations

The retrospective design does not allow for verification of the documented data. Whether the presence of FGM/C was detected but not documented cannot be assured retrospectively. To produce the most accurate analysis, the country of origin was prioritized over the country of citizenship. This decision was made under the assumption that the prevalence figures of the country of birth were more accurate than those of the country of citizenship. In the estimation of FGM/C prevalence in our population we assumed that the prevalence figures published by UNICEF could be applied to migrant women living in Austria. However, studies suggest education, cultural adaptation, length of residence in a country where FGM/C is illegal and being part of a new generation or community reduce migrants’ support for the practice [41]. Thus, the estimated prevalence of our study population does probably not accurately reflect reality, but rather represents a worst-case scenario.

Our results indicate an unmet need for specific obstetrical and gynecological care for women after FGM/C in our area. They highlight the lack of awareness by healthcare personnel and the need for specific training. Because this issue affects the whole family including the possible female newborn, an interdisciplinary approach with pediatricians, psychologists as well as social workers should be favored [1, 4, 57].

This underlines the suggestion of the European Institute for Gender Equality for counseling infrastructure (gynecologists, obstetricians, pediatricians, psychiatrists, psychologists, social workers, interpreters) in every Austrian state [29]. Girls and women who have undergone female genital mutilation as well as their partners, children and relatives should have the opportunity to receive the best possible counseling.

## Data Availability

Data was retrospectively collected from the hospital and medical records of the Medical University of Graz, Department if Gynecology and obstetrics. The datasets generated and analyzed during the current study are not publicly available due to medical data protection but are available from the corresponding author on reasonable request.
